# Sinus bradycardia after intravenous methylprednisolone pulse therapy in patients with thyroid-associated ophthalmopathy

**DOI:** 10.3389/fphar.2026.1903935

**Published:** 2026-08-28

**Authors:** Yaning Zhang, Tiantian Gao, Yahui Zhang, Wen Zhang, Hengcai Yu

**Affiliations:** Department of Pharmacy, Shandong Provincial Hospital Affiliated to Shandong First Medical University, Jinan, China

**Keywords:** adverse events, bradycardia, methylprednisolone, pulse therapy, thyroid-associated ophthalmopathy

## Abstract

**Background:**

Intravenous methylprednisolone pulse (IVMP) therapy is the first-line treatment for active moderate-to-severe and very severe thyroid-associated ophthalmopathy (TAO). IVMP-induced sinus bradycardia has not been previously reported in patients with TAO in the published English-language literature. This study aimed to determine the prevalence and characteristics of IVMP-induced bradycardia in patients with TAO and to analyze the associated factors.

**Methods:**

This retrospective cohort study was conducted on 166 patients with TAO who received IVMP therapy at Shandong Provincial Hospital between January 2023 and March 2026. Clinical data were collected during hospitalization. Sinus bradycardia was defined as a resting heart rate below 60 beats/min. The prevalence and features of bradycardia were evaluated. Furthermore, multivariable Cox regression and sensitivity analyses were performed to identify independently associated factors.

**Results:**

Bradycardia occurred in 28.9% (48/166) of individuals receiving IVMP therapy. Among those with bradycardia, 79.2% (38/48) experienced their first episode within 3 days of initiating treatment, and 12.5% (6/48) had two episodes during IVMP. The mean lowest heart rate of first bradycardia was 46.9 beats/min, representing a 42.1% decrease from baseline (80.9 beats/min). The first episodes of bradycardia lasted 2.8 days on average and within 4 days mostly. No clinically significant decrease in diastolic blood pressure was observed during the first bradycardia episodes, despite statistical significance. Multivariable Cox regression analysis identified five factors independently associated with bradycardia. Heart rate (HR 0.971, 95% CI 0.945–0.998, *p* < 0.05), BMI (HR 0.908, 95% CI 0.825–0.999, *p* < 0.05), and periorbital edema (HR 0.403, 95% CI 0.219–0.742, *p* < 0.05) were associated with a lower likelihood of bradycardia, whereas cardiac disease (HR 2.454, 95% CI 1.105–5.450, *p* < 0.05) and TSH (HR 1.028, 95% CI 1.006–1.050, *p* < 0.05) were associated with a higher likelihood.

**Conclusion:**

Bradycardia commonly occurred during IVMP therapy in individuals with TAO. Heart rate, BMI, and periorbital edema were significantly and inversely associated with sinus bradycardia, whereas cardiac disease and TSH showed significant positive associations. These findings highlight the importance of cardiac monitoring in individuals at high risk of bradycardia during IVMP treatment.

## 1 Introduction

Thyroid-associated ophthalmopathy (TAO), also known as Graves’ ophthalmopathy, is an organ-specific autoimmune disease closely linked to thyroid disorders. It is the most common orbital disease in adults and represents the primary extrathyroidal manifestation of Graves’ disease (Bartalena et al., 2021). TAO can lead to proptosis, extraocular muscle involvement, and visual disturbances—including diplopia, optic neuropathy, and, in severe cases, blindness—substantially impairing quality of life (Ma et al., 2024). Because the pathogenesis of TAO remains incompletely understood and its clinical presentation is complex, treatment strategies remain heterogeneous and incompletely standardized. Current options include pharmacotherapy, orbital radiotherapy, and surgical interventions. Intravenous glucocorticoid pulse therapy is recommended as the first-line treatment for active, moderate-to-severe, and very severe TAO. Its therapeutic effects are attributed to the inhibition of cytokine production, orbital fibroblast proliferation, and glycosaminoglycan synthesis or release (Oculoplastic and Orbital Disease Group of Chinese Ophthalmological Society of Ch inese Medical Association, 2022). Approximately 70%–80% of patients show clinical improvement within 3–6 months of treatment, including reduced proptosis, alleviated diplopia, improved visual acuity, and a significant decrease in the clinical activity score (CAS) (Bartalena et al., 2021; Oculoplastic and Orbital Disease Group of Chinese Ophthalmological Society of Ch inese Medical Association, 2022).

Common adverse events associated with intravenous glucocorticoid pulse therapy include infection, hyperglycemia, hyperlipidemia, and hypertension (Bartalena et al., 2021; Deng et al., 2023), whereas bradycardia is generally considered rare. Existing reports of glucocorticoid-induced sinus bradycardia primarily involve individuals with multiple sclerosis or rheumatic diseases (Vasheghani-Farahani et al., 2011; Nagakura et al., 2017; Beyan et al., 2004; Ohshima et al., 2017; Ogiwara et al., 2024; Sodero et al., 2019; Jain et al., 2005). To date, no cases of sinus bradycardia following intravenous glucocorticoid pulse therapy have been reported in patients with TAO in the published English-language literature. This study aimed to examine the frequency and characteristics of intravenous methylprednisolone pulse (IVMP)-induced sinus bradycardia in individuals with TAO and to evaluate the correlation between the clinical course of the disease and the occurrence of sinus bradycardia through a retrospective analysis of cases at our hospital.

## 2 Materials and methods

This study reviewed the electronic medical records of all patients with TAO who received IVMP therapy and were admitted to the Endocrinology Department of Shandong Provincial Hospital Affiliated to Shandong First Medical University, between 1 January 2023, and 31 March 2026. The following groups were excluded: patients with TAO accompanied by pre-existing sinus bradycardia; those who discontinued IVMP therapy midway; and those with seriously incomplete clinical data. This study was reviewed and approved by Medical Ethics Committee of Shandong Provincial Hospital Affiliated to Shandong First Medical University (SWYX: NO. 2024-453).

Clinical data were collected through the hospital information system, including demographic and anthropometric characteristics (sex, age, height, weight, body mass index (BMI), temperature, smoking and alcohol status, and hospital stay), thyroid-related parameters (history of thyroid disease, current thyroid function, TAO duration, and CAS), laboratory measures (electrolytes, liver and kidney function), treatment details (IVMP regimen and β-blocker use), ocular findings, concomitant diseases, and pre-IVMP vital signs (BP and HR).

Clinical data were collected through the hospital information system. The following variables were extracted (Bartalena et al., 2021): demographic and anthropometric characteristics, including sex, age, height, weight, body mass index (BMI), body temperature, smoking and alcohol history, and length of hospital stay (Ma et al., 2024); thyroid-related parameters, including history of thyroid disease, current thyroid function, duration of TAO, and CAS (Oculoplastic and Orbital Disease Group of Chinese Ophthalmological Society of Ch inese Medical Association, 2022); laboratory indices, including serum electrolytes and liver and kidney function (Deng et al., 2023); treatment-related variables, including the frequency, cumulative dose, and duration of IVMP therapy, and β-blocker use (Vasheghani-Farahani et al., 2011); ocular examination findings (Nagakura et al., 2017); concomitant diseases; and (Beyan et al., 2004) pre-IVMP vital signs, including blood pressure (BP) and heart rate (HR). All patients receiving IVMP therapy were placed under continuous bedside electrocardiographic monitoring throughout the treatment period using a Mindray ePM 10C patient monitor (Shenzhen Mindray Bio-Medical Electronics Co., Ltd., China). The monitoring data were continuously displayed and automatically recorded, enabling timely detection of bradycardic events. Sinus bradycardia was defined as a resting HR below 60 beats/min (Kusumoto et al., 2019). Based on this criterion, individuals were divided into bradycardia and non-bradycardia groups.

All statistical analyses were performed using SPSS version 27.0. The proportion of missing values ranged from 0.6% (serum potassium and magnesium) to 9.1% (TPOAb) across all clinical and laboratory variables. To preserve statistical power and minimize potential bias, multiple imputation was performed to generate complete datasets for all subsequent analyses. The Kolmogorov–Smirnov test was used to assess the normality of continuous variables. For normally distributed data, differences were analyzed using the student’s t-test; for non-normally distributed data, the Mann–Whitney U test was applied. Categorical variables were analyzed using the chi-square test.

Variables with a univariate *P*-value < 0.05 were considered for further selection, with additional considerations based on clinical relevance and literature evidence. Key confounders, particularly thyroid functional status, were also evaluated for inclusion due to its established inverse association with HR—hyperthyroidism accelerates HR, whereas hypothyroidism slows it. Given that only 48 bradycardia events were observed, the number of covariates that could be reliably entered into the multivariable model was constrained to prevent overfitting. Through this process, five variables were ultimately entered into the multivariable Cox proportional hazards model to identify factors associated with the risk of sinus bradycardia. Results are presented as hazard ratios (HRs) with 95% confidence intervals (CIs). Kaplan-Meier analysis was performed to estimate the cumulative incidence of bradycardia events during follow-up. A two-sided *P* < 0.05 was considered statistically significant.

To comprehensively assess model robustness, sensitivity analyses were performed using nested Cox regression models, supplemented by collinearity diagnostics. Three hierarchical models were constructed: Model 1 (core model, adjusted for baseline HR, cardiac disease, periorbital edema, TSH and BMI), Model 2 (clinically adjusted, Model 1 + additionally adjusted for continuous CAS), and Model 3 (extended sensitivity model, Model 2 + additionally adjusted for β-blocker use and cumulative IVMP dose). Robustness was evaluated by comparing changes in the hazard ratios (HRs) of the core variables across the three models; a change of less than 10% in the HR was considered indicative of robustness against additional confounders. Multicollinearity was assessed using the variance inflation factor (VIF), with VIF ≤5 indicating no significant multicollinearity. In addition, model discrimination was assessed using Harrell’s C-index. The C-index ranges from 0.5 to 1.0, with 0.5 indicating no predictive ability and 1.0 indicating perfect prediction.

## 3 Results

### 3.1 Comparative basic characteristics of the study population

**FIGURE 1:**
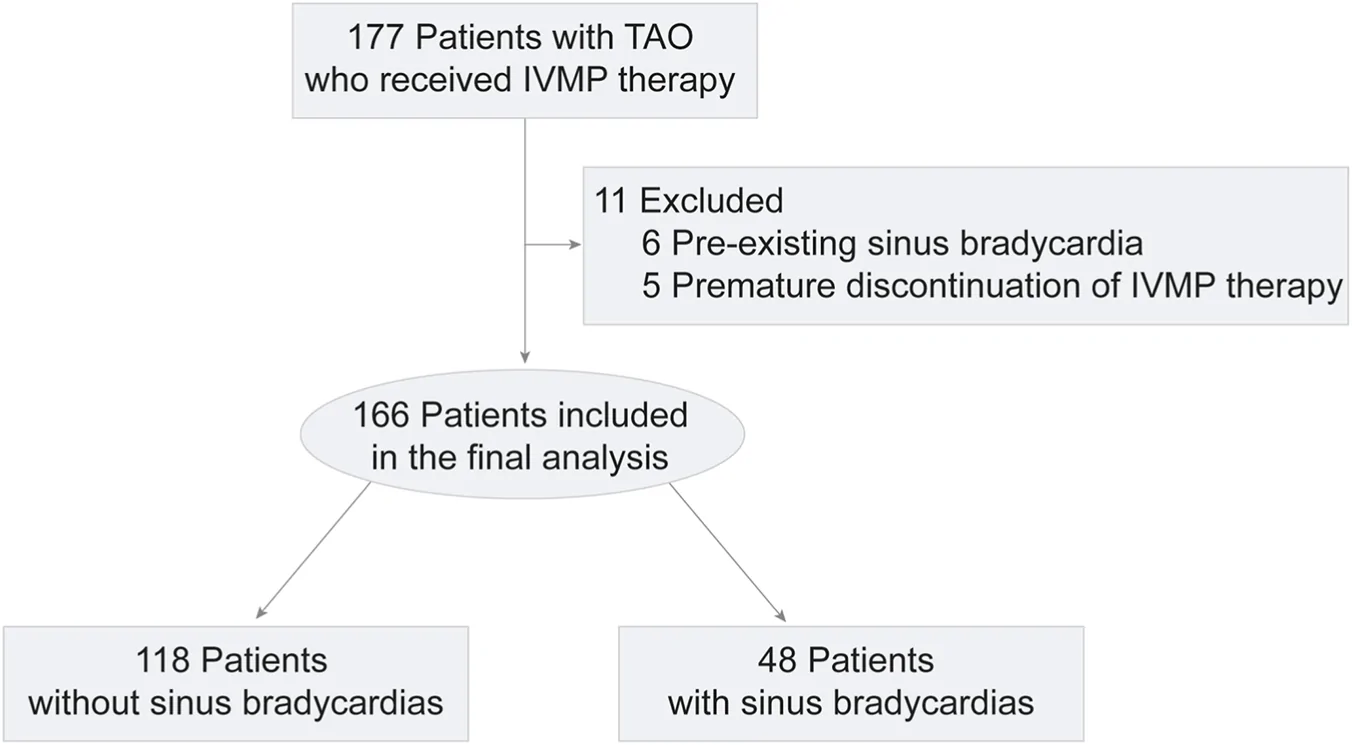
Flow of patients through the study. TAO, thyroid-associated ophthalmopathy; IVMP, intravenous methylprednisolone pulse.

This retrospective study reviewed 177 patients with TAO who received IVMP therapy in the Department of Endocrinology at Shandong Provincial Hospital Affiliated to Shandong First Medical University, between 1 January 2023, and 31 March 2026. Individuals with pre-existing sinus bradycardia, premature discontinuation of pulse therapy, or incomplete clinical data were excluded. A total of 166 patients were included in the final analysis. Among them, 48 developed sinus bradycardias, corresponding to a cumulative incidence of 28.9% (Figure 1).

The mean age was comparable between the two groups (45.85 years vs. 47.88 years, *p* > 0.05). Females accounted for 60.4% and 44.9% of each group, respectively. Significant differences were observed between the groups in BMI, HR, systolic BP, serum magnesium, hemoglobin levels, AST, comorbidity including cardiac disease, dyslipidemia and hypertension (Table 1). Ocular symptoms such as lacrimation and periorbital edema, also differed significantly (Table 2).

**TABLE 1:** Characteristics of the study population.

Characteristics	Non-bradycardia group (n = 118)	Bradycardia group (n = 48)	χ^2^/t/Z	*P* value
Basic information				
Gender (female)	53 (44.9%)	29 (60.4%)	3.280^a^	0.070
Age (year)	47.88 (12.68)	45.85 (12.54)	0.937^b^	0.350
Height (m)	1.67 (1.60–1.72)	1.65 (1.60–1.73)	−0.427^c^	0.670
Weight (kg)	70.00 (60.75–80.00)	65.00 (59.25–73.75)	−1.773^c^	0.076
BMI (kg/m^2^)	25.44 (3.39)	24.17 (3.00)	2.259^b^	0.025*
Body temperature (°C)	36.40 (36.20–36.80)	36.30 (36.20–36.56)	−1.126^c^	0.260
HR (beats/min)	85.72 (12.32)	80.9 (10.90)	2.362^b^	0.019*
Systolic BP (mmHg)	129.19 (16.41)	123.04 (12.84)	2.323^b^	0.021*
Diastolic BP (mmHg)	82.14 (12.05)	79.52 (9.17)	1.514^b^	0.133
Smoking history	24 (20.3%)	4 (8.3%)	3.507^a^	0.061
Drinking history	25 (21.2%)	7 (14.6%)	0.956^a^	0.328
In-hospital patient days	16 (14–18)	16 (15–17)	−0.205^c^	0.838
History of thyroid disease	​	​	3.633^a^	0.458
Graves’ hyperthyroidism	98 (83.1%)	41 (85.4%)	0.140^a^	0.708
Primary hypothyroidism	12 (10.2%)	6 (12.5%)	0.192^a^	0.662
Thyroid cancer	2 (1.7%)	0 (0.0%)	0.015^a^	0.902
Benign thyroid nodule	3 (2.5%)	1 (2.1%)	0.000^a^	1.000
Other diseases	3 (2.5%)	0 (0.0%)	0.223^a^	0.637
Comorbidity				
Cardiac disease	5 (4.2%)	9 (18.8%)	7.521^a^	0.006**
Atrial fibrillation	1 (0.8%)	0 (0.0%)	0.000^a^	1.000
Nodal tachycardia	1 (0.8%)	0 (0.0%)	0.000^a^	1.000
Atrioventricular block	1 (0.8%)	3 (6.3%)	2.249^a^	0.134
Premature ventricular contraction	0 (0.0%)	1 (2.1%)	0.218^a^	0.641
Coronary heart disease	2 (1.7%)	4 (8.3%)	2.621	0.105
Rheumatic heart disease	0 (0.0%)	1 (2.1%)	0.218	0.641
Dyslipidemia	62 (52.5%)	17 (35.4%)	4.012^a^	0.045*
Diabetes	22 (18.6%)	5 (10.4%)	1.696^a^	0.193
Hypertension	29 (24.6%)	5 (10.4%)	4.200^a^	0.040*
Current thyroid function				
FT3 (pmol/L)	4.72 (4.09–5.76)	4.34 (3.73–5.65)	−1.546^c^	0.122
FT4 (pmol/L)	13.97 (11.78–17.08)	13.44 (10.32–17.28)	−0.531^c^	0.596
TSH (μIU/mL)	0.42 (0.01–2.09)	1.12 (0.04–3.94)	−1.668^c^	0.095
TRAb (+)	99 (83.9%)	38 (79.2%)	0.530^a^	0.467
TPOAb (+)	42 (35.6%)	15 (31.3%)	0.285^a^	0.593
Duration of TAO (month)	6.0 (4.0–12.0)	8.5 (4.0–12.0)	−1.066^c^	0.286
Serum electrolytes levels				
Potassium (mmol/L)	3.91 (0.27)	3.97 (0.32)	−1.090^b^	0.277
Calcium (mmol/L)	2.35 (0.10)	2.34 (0.09)	0.723^b^	0.471
Sodium (mmol/L)	139.76 (1.93)	139.25 (1.96)	1.539^b^	0.126
Magnesium (mmol/L)	0.87 (0.06)	0.89 (0.04)	−2.254^b^	0.026*
Chloride (mmol/L)	104.98 (2.43)	104.71 (2.28)	0.671^b^	0.503
Albumin (g/L)	42.15 (3.17)	42.74 (2.74)	−1.132^b^	0.259
Hemoglobin (g/L)	139.67 (14.44)	133.90 (17.18)	2.208^b^	0.029*
Liver and kidney function				
ALT (U/L)	19.00 (15.00–26.25)	18.50 (11.25–25.50)	−1.013^c^	0.311
AST (U/L)	19.00 (17.00–24.00)	21.00 (18.00–25.75)	−1.992^c^	0.046*
eGFR (mL/min)	112.72 (14.72)	111.57 (16.84)	0.439^b^	0.661
Number of IVMP this admission	​	​	0.334^a^	0.953
First	87 (73.7%)	35 (72.9%)	0.012^a^	0.914
Second	26 (22.0%)	10 (20.8%)	0.029^a^	0.865
Third	3 (2.5%)	2 (4.2%)	0.003^a^	0.957
Fourth	2 (1.7%)	1 (2.1%)	0.000^a^	1.000
Cumulative IVMP dose this admission (g)				
1.5–3.0	23 (19.5%)	7 (14.6%)	0.555^a^	0.456
3.1–4.5	95 (80.5%)	41 (85.4%)	0.555^a^	0.456
Duration of IVMP (d)	15.00 (12.00–15.00)	15.00 (13.00–15.00)	−1.234^c^	0.217
Concomitant β-receptor blocker with IVMP	31 (26.3%)	14 (29.2%)	0.145^a^	0.704

BMI, body mass index; HR, heart rate; BP, blood pressure; FT3, free triiodothyronine; FT4, free thyroxine; TSH, thyroid stimulating hormone; TRAb, thyrotropin receptor antibodies; TPOAb, thyroid peroxidase antibody; TAO, thyroid-associated ophthalmopathy; ALT, alanine aminotransferase; AST, aspartate aminotransferase; eGFR, estimated glomerular filtration rate; IVMP, intravenous methylprednisolone pulse.

Categorical variables are summarized as “n (%)”, continuous variables with a normal distribution are summarized as “mean (standard deviation)”, and continuous variables without a normal distribution are summarized as “median (interquartile range)”.

^a^χ2 value of chi-square test;

^b^t value of Student’s t-test;

^c^Z value of Mann-Whitney U test.

*p < 0.05,

**p < 0.01, between bradycardia group and non-bradycardia group.

**TABLE 2:** Characteristics of patients’ ocular manifestations before IVMP therapy.

Characteristics	Non-bradycardia group (n = 118)	Bradycardia group (n = 48)	χ^2^/t/Z	*P* value
Eye symptoms				
Photophobia	57 (48.3%)	20 (41.7%)	0.605^a^	0.437
Lacrimation	81 (68.6%)	25 (52.1%)	4.054^a^	0.044*
Periorbital edema	97 (82.2%)	31 (64.6%)	6.001^a^	0.014*
Gritty sensation	9 (7.6%)	5 (10.4%)	0.077^a^	0.781
Ocular pain	28 (23.7%)	12 (25.0%)	0.030^a^	0.862
Visual acuity decline	52 (44.1%)	18 (37.5%)	0.604^a^	0.437
Diplopia	64 (54.2%)	22 (45.8%)	0.965^a^	0.326
Proptosis (mm)				
Right eye	19.92 (3.40)	20.01 (2.78)	−0.187^b^	0.852
Left eye	19.99 (3.16)	19.73 (2.56)	0.558^b^	0.578
CAS	​	​	2.182^a^	0.336
CAS <3	16 (13.6%)	11 (22.9%)	2.194^a^	0.139
3 ≤ CAS ≤5	95 (80.5%)	35 (72.9%)	1.158^a^	0.282
6 ≤ CAS ≤7	7 (5.9%)	2 (4.2%)	0.006^a^	0.938
ocular signs				
Joffroy sign	10 (8.5%)	5 (10.4%)	0.009^a^	0.923
Mobius sign	19 (16.1%)	4 (8.3%)	1.725^a^	0.189
vonGraefe sign	12 (10.2%)	4 (8.3%)	0.005^a^	0.941
Stellwag sign	10 (8.5%)	6 (12.5%)	0.257^a^	0.612

IVMP, intravenous methylprednisolone pulse; CAS, clinical activity score.

Categorical variables are summarized as “n (%)”, and continuous variables with a normal distribution are summarized as “mean (standard deviation)”.

^a^χ^2^ value of chi-square test;

^b^t value of Student’s t-test.

*p < 0.05, between bradycardia group and non-bradycardia group.

No statistically significant differences were observed between the two groups in terms of other baseline information, including demographic and anthropometric characteristics, thyroid-related parameters (history of thyroid disease, current thyroid function, and TAO duration), comorbidities, laboratory indices (serum electrolytes, liver and kidney function), β-blocker use, ocular symptoms, and IVMP treatment parameters (frequency, cumulative dose, and duration). The standard IVMP regimen at our hospital consists of 0.5 g/day for the first 5 days, 0.3 g/day for the next 5 days, and 0.1 g/day for the final 5 days, with adjustments made in some cases based on individual clinical conditions. The characteristics of the study population are summarized in Table 1, and ocular features are presented in Table 2.

### 3.2 Characteristics and management of bradycardia episodes

**FIGURE 2:**
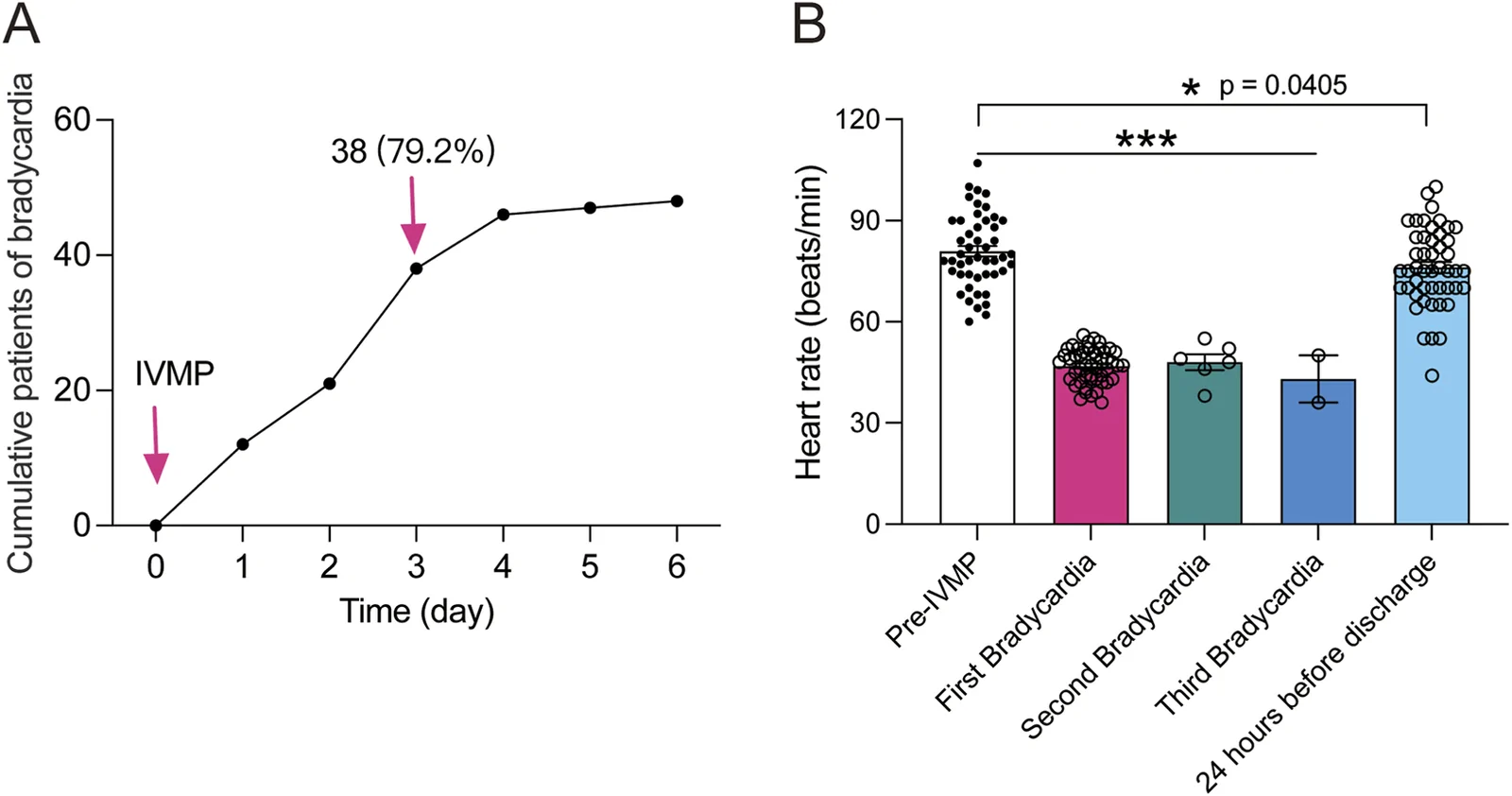
Time to onset and comparison of heart rate (HR) in individuals with bradycardia following intravenous methylprednisolone pulse (IVMP) therapy. **(A)** Cumulative patients who developed bradycardia after initiating IVMP therapy (day 0). Of the 48 cases, 79.2% (38/48) experienced bradycardia within 72 h (3 days) of treatment initiation. **(B)** Heart rate before and after IVMP therapy in patients who developed bradycardia. Data represent the lowest HR during bradycardia episodes. n = 48 for the Pre-IVMP, First Bradycardia, and 24 h before discharge groups, n = 6 for the Second Bradycardia group, n = 2 for the Third Bradycardia group. IVMP, intravenous methylprednisolone pulse; HR, heart rate. HR data are presented as mean (standard error). ***p < 0.001, *p < 0.05, compared with pre-IVMP heart rate.

**FIGURE 3:**
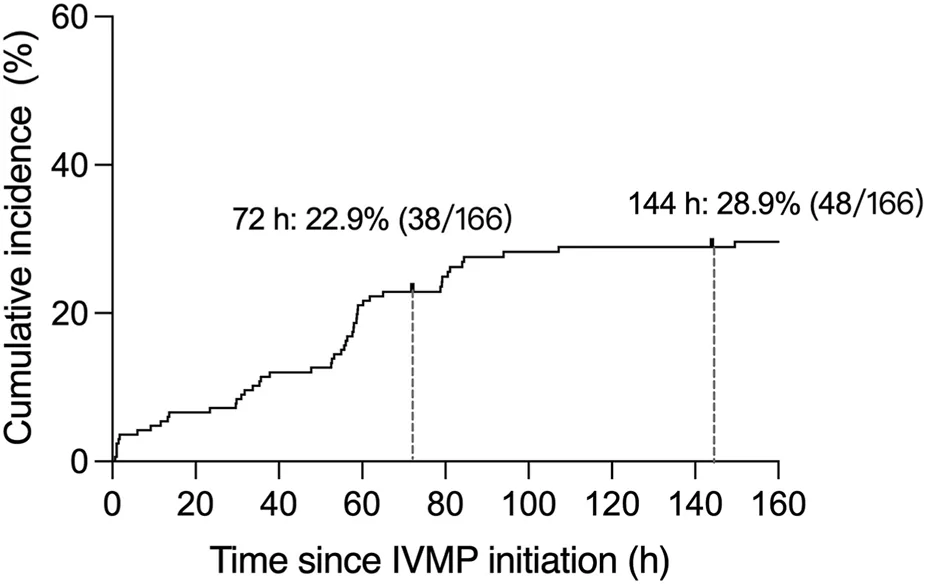
Kaplan-Meier curve for bradycardia. Development of bradycardia was defined as an event occurrence. The cumulative incidence of bradycardia reached 22.9% within the first 72 h following IVMP therapy. IVMP, intravenous methylprednisolone pulse.

**FIGURE 4:**
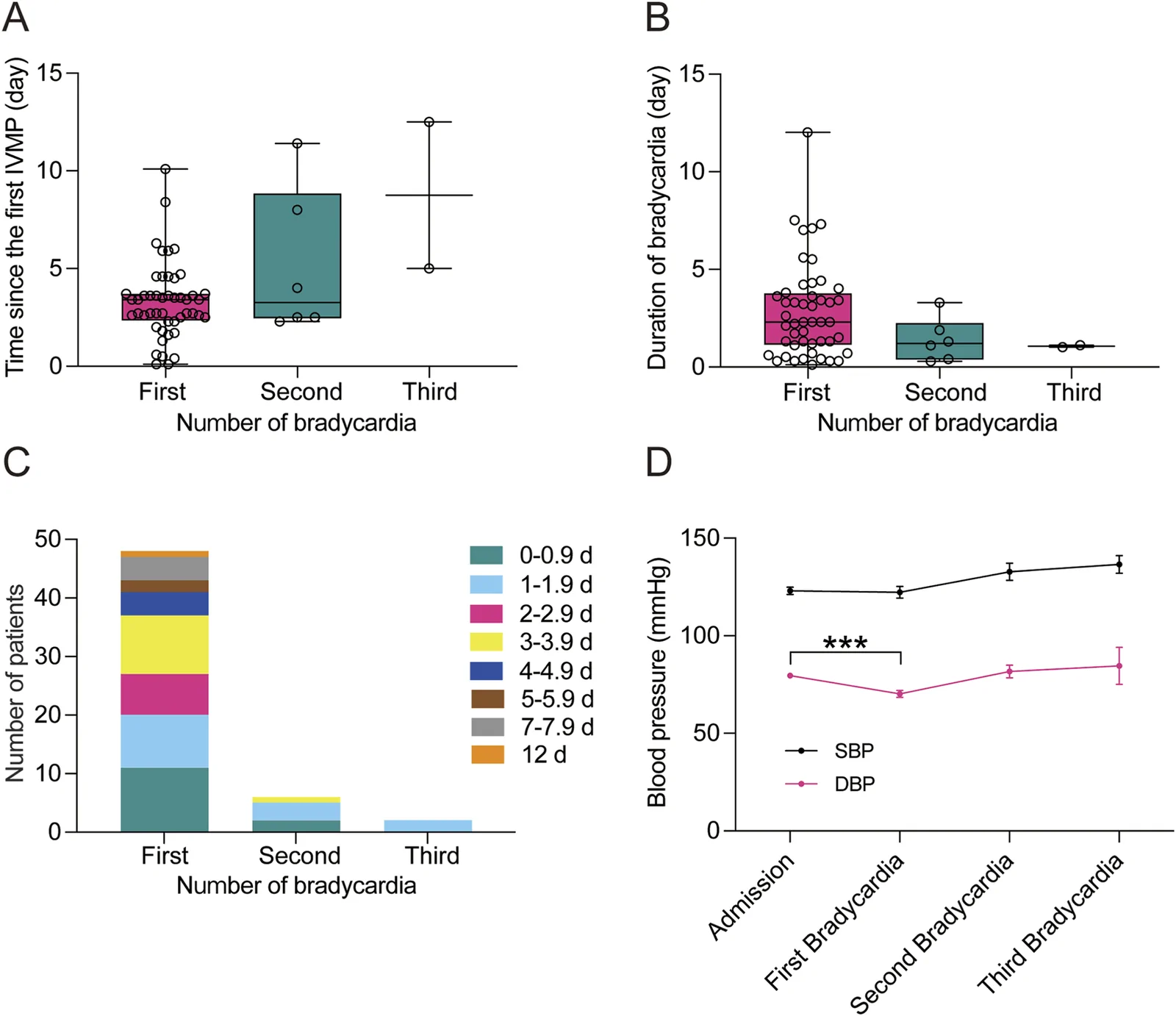
Characteristics of individuals with bradycardia after IVMP therapy **(A)** time from the first injection to the lowest HR during bradycardia **(B)** duration of each bradycardia episode **(C)** total number of patients and distribution by bradycardia episode frequency **(D)** blood pressure (BP) at admission and at the time of the lowest heart rate during bradycardia. n = 48 for the Pre-IVMP, First Bradycardia, n = 6 for the Second Bradycardia group, n = 2 for the Third Bradycardia group. IVMP, intravenous methylprednisolone pulse; BP, blood pressure; SBP, systolic blood pressure; DBP, diastolic blood pressure. In panels **(A)** and **(B)**, box plots extend from the 25th to the 75th percentiles, with the median indicated by the internal line. In panels **(D)**, BP values are presented as mean (standard error). ***p < 0.001, comparing the First Bradycardia group with the Admission group.

We analyzed the features of bradycardia induced by IVMP therapy in patients with TAO. Bradycardia occurred within 3 days of initiating treatment in 79.2% (38/48) of cases, whereas the remainder developed bradycardia within 6 days of starting therapy (Figure 2A). The Kaplan-Meier curve showed a rapid increase in cumulative bradycardia incidence within the first 72 h (reaching ∼28%), followed by a relative plateau (Figure 3). The mean lowest HR of first bradycardia observed after IVMP therapy was 46.9 beats/min, representing a 42.1% reduction compared with the pre-treatment HR of 80.9 beats/min (Figure 2B). The lowest point of the first bradycardia occurred, on average, 3.3 days after the first injection (Figure 4A). By the time of discharge, the average HR in the bradycardia group had increased to 76.1 beats/min (Figure 2B), with or without intervention.

Approximately 12.5% (6/48) of individuals in the bradycardia group experienced two episodes, and 4.2% (2/48) individuals experienced three episodes (Figures 2B, 4A,B). The mean duration of each episode tended to decrease as the number of episodes increased, from 2.8 days for the first episode to 1.4 days for the second and 1.1 days for the third (Figure 4B). Most episodes lasted less than 4 days, with durations ranging from 1.3 h to 12 days (Figure 4C). Diastolic BP during first bradycardia episode showed statistically significant decrease compared with values before onset (Figure 4D).

Among the 48 patients who developed bradycardia (14 with HR 50–60 beats/min, 28 with 40–50 beats/min, and 6 with ≤40 beats/min), all underwent continuous bedside electrocardiographic monitoring using a Mindray ePM 10C patient monitor. Among the four symptomatic patients, one presented with precordial pain, wheezing, and tightness, leading to metoprolol discontinuation; one had chest discomfort and received supplemental trimetazidine; one experienced chest distress and was treated with both trimetazidine and isosorbide mononitrate; and the remaining one developed limb weakness and dizziness, for which potassium chloride injection plus potassium citrate was administered (Table 3). The remaining cases improved spontaneously with close monitoring and without additional intervention.

**TABLE 3:** Stratification and interventions in 48 patients with bradycardia.

Groups	Total (n = 48)	Symptomatic bradycardia (n = 4)	Clinical intervention (n = 4)
50 < HR < 60 No. (%), beats/min	14 (29.17)	0 (0)	0 (0)
40 < HR ≤ 50 No. (%), beats/min	28 (58.33)	3 (75.0) Patient 1 chest distress Patient 2 weakness of limbs, dizziness Patient 3 chest discomfort	3 (75.0) Patient 1 Add trimetazidine, isosorbide mononitrate Patient 2 Add potassium chloride injection, potassium citrate Patient 3 Add trimetazidine
HR ≤ 40 No. (%), beats/min	6 (12.5)	1 (25.0) Patient 4 precordial pain, wheezing and tightness	1 (25.0) Patient 4 discontinue metoprolol

Data are presented as “n (%)”. Symptoms and corresponding treatments for the 4 symptomatic bradycardia patients are shown in the table. HR, heart rate.

### 3.3 Analysis of factors associated with bradycardia

To identify factors associated with bradycardia after IVMP therapy in TAO patients, we constructed a multivariable Cox regression model including heart rate, BMI, periorbital edema, TSH, and concomitant cardiac disease as covariates. As shown in Table 4, heart rate (HR 0.971, 95% CI 0.945–0.998, *p* < 0.05), BMI (HR 0.908, 95% CI 0.825–0.999, *p* < 0.05), and periorbital edema (HR 0.403, 95% CI 0.219–0.742, *p* < 0.05) were independently associated with a lower likelihood of bradycardia. Conversely, concomitant cardiac disease (HR 2.454, 95% CI 1.105–5.450, *p* < 0.05) and higher TSH (HR 1.028, 95% CI 1.006–1.050, *p* < 0.05) were independently associated with a higher likelihood of bradycardia.

**TABLE 4:** Multivariable Cox regression and sensitivity analyses for factors associated with sinus bradycardia after IVMP therapy.

Variables	The core model			Sensitivity analysis					
	Model 1			Model 2			Model 3		
	β (SE)	Adjusted HR (95% CI)	*P* value	Adjusted HR (95% CI)	*P* value	% Change in HR	Adjusted HR (95% CI)	*P* value	*%* Change in HR
Heart rate (beats/min)	−0.029 (0.014)	0.971 (0.945–0.998)	0.037*	0.971 (0.944–0.999)	0.040*	0%	0.971 (0.944–0.998)	0.038*	0%
Cardiac disease	0.898 (0.407)	2.454 (1.105–5.450)	0.027*	2.467 (1.105–5.506)	0.028*	0.53%	2.440 (1.093–5.447)	0.029*	−0.57%
Periorbital edema	−0.909 (0.311)	0.403 (0.219–0.742)	0.004*	0.407 (0.214–0.774)	0.006*	0.99%	0.407 (0.213–0.779)	0.007*	0.99%
TSH (μIU/mL)	0.027 (0.011)	1.028 (1.006–1.050)	0.012*	1.028 (1.006–1.050)	0.012*	0%	1.028 (1.006–1.050)	0.012*	0%
BMI (kg/m^2^)	−0.096 (0.049)	0.908 (0.825–0.999)	0.049*	0.908 (0.825–0.999)	0.049*	0%	0.907 (0.824–0.999)	0.048*	−0.11%

Model 1: Adjusted for heart rate, cardiac disease, periorbital edema, TSH, and BMI.

Model 2: Model 1 + additionally adjusted for continuous CAS.

Model 3: Model 2 + additionally adjusted for β-blocker use (yes/no) and cumulative IVMP, dose (g).

IVMP, intravenous methylprednisolone; BMI, body mass index; TSH, thyroid stimulating hormone; HR, hazard ratio; CI, confidence interval; SE, standard error; CAS, clinical activity score.

% Change in HR: Percentage change in hazard ratio compared to the first model. A change > ± 10% was considered indicative of meaningful confounding. *p < 0.05, represents significantly associated with bradycardia.

Sensitivity analyses were further performed to assess the robustness of our findings. As shown in Table 4, three nested Cox regression models were constructed with stepwise adjustment for potential confounders: Model 1 adjusted for HR, cardiac disease, periorbital edema, TSH, and BMI; Model 2 further adjusted for continuous CAS; and Model 3 additionally adjusted for β-blocker use and cumulative IVMP dose. Across these models, the HRs for the primary covariates remained stable, with changes of less than 1% relative to the core model, indicating that the observed associations were not substantially influenced by the inclusion of additional covariates. Moreover, variance inflation factor (VIF) values were all below 5, suggesting no significant multicollinearity (Table 5). Taken together, these results support the robustness of our multivariable Cox regression model. In terms of discriminative performance, time-dependent ROC analysis yielded a Harrell’s C-index of 0.605 (95% CI 0.497–0.712, *p* = 0.035), indicating modest individual-level predictive ability.

**TABLE 5:** Collinearity diagnostics for variables included in the final multivariable Cox regression model.

Variables	Tolerance	VIF
Heart rate (beats/min)	0.961	1.040
Cardiac disease	0.940	1.064
Periorbital edema	0.978	1.022
BMI (kg/m^2^)	0.986	1.014
TSH (μIU/mL)	0.931	1.074

VIF, variance inflation factor; BMI, body mass index; TSH, thyroid-stimulating hormone.

## 4 Discussion

### 4.1 Incidence of bradycardia

The cumulative prevalence of bradycardia in our study (28.9%) was comparable to that previously reported in corticosteroids-treated pemphigus patients (30%) (Jain et al., 2005). However, it was considerably lower than the rates observed in multiple sclerosis (41.9%) (Vasheghani-Farahani et al., 2011), Kawasaki disease (79.1% and 82.0%) (Nagakura et al., 2017; Miura et al., 2005). One possible explanation for this discrepancy is that autonomic dysfunction is a common feature of multiple sclerosis and Kawasaki disease but not typically seen in TAO or pemphigus. Vasheghani-Farahani et al. noted that diseases involving autonomic dysfunction are more likely to trigger bradycardia during glucocorticoid pulse therapy (Vasheghani-Farahani et al., 2011). Another possible explanation for the variation in reported prevalence is the differing definitions of bradycardia. In this study, bradycardia was defined as a HR below 60 beats/min, a threshold appropriate for adults. In contrast, some studies—particularly those involving children—used definitions based on HR percentiles from pediatric reference charts (Nagakura et al., 2017).

### 4.2 Characteristics of bradycardia episodes

In our retrospective study, bradycardia occurred within 3 days of treatment initiation in 79.2% of affected patients, with a median onset time of 2.3 days. This was consistent with the median onset of 2.6 days reported in approximately half of children with Kawasaki disease receiving prednisolone (Nagakura et al., 2017). The mean lowest HR during first bradycardia episode in this study was 46.9 beats/min, representing a 42.1% decrease from the pre-treatment value of 80.9 beats/min. A similar pattern was observed in a white woman with inflammatory myelitis, in whom HR declined from 76 to 37 beats/min (a 51.3% reduction) following high-dose methylprednisolone therapy (Sodero et al., 2019). Likewise, a 40% reduction in HR (from 70 to 42 beats/min) was reported 36 h after administration of 52 mg/day methylprednisolone in a patient with Takayasu’s arteritis (Cansu et al., 2018). In the current study, only 8.33% (4/48) of patients with bradycardia required intervention, whereas the remainder improved spontaneously, consistent with prior reports indicating that bradycardia often resolves within a few days after discontinuing treatment (Sodero et al., 2019; Cansu et al., 2018).

Most individuals in this study received a 15-day IVMP regimen of 0.5 g/day for 5 days, 0.3 g/day for the next 5 days, and 0.1 g/day for the final 5 days, except for a few who required dose reduction owing to intolerance. Among those who developed bradycardia, 12.5% and 4.2% experienced two and three episodes, respectively. Most previous studies have reported the duration of a single bradycardia episode—ranging from 4 h to 11 days (Beyan et al., 2004; Taylor and Gaco, 2013). Consistently with the above, bradycardia in this study occurred ranging from 1.3 h to 12 days. Furthermore, as the number of episodes increased, both the number of affected individuals and the mean duration of each episode decreased. This pattern may be attributed to increased tolerance to IVMP therapy over time or to the gradual reduction in the daily methylprednisolone dose.

### 4.3 Factors associated with bradycardia

In this study, multivariable Cox regression analysis revealed that HR and BMI were independently and inversely associated with the cumulative incidence of sinus bradycardia. Previous studies have identified low baseline HR and low BMI as potential associated factors for bradycardia. Limmroth (Limmroth et al., 2020) reported that a low pre-treatment HR may predispose patients to bradycardia, while Kim (Kim and Ahn, 2022) identified a lower baseline HR as a significant predictor of dexmedetomidine-associated bradycardia during spinal anesthesia. In addition, Goleva (Goleva et al., 2016) observed corticosteroid pharmacokinetic abnormalities in overweight and obese patients with corticosteroid-resistant asthma, with rapid clearance potentially contributing to suboptimal therapeutic responses. In line with these observations, our study demonstrated that patients who developed sinus bradycardia following IVMP therapy had significantly lower baseline HR and BMI compared to those who did not. Although these values remained within the clinically normal range, even relatively lower normal values may predispose patients to bradycardia during high-dose corticosteroid therapy.

Notably, periorbital edema—a hallmark of active TAO—was inversely associated with the cumulative occurrence of bradycardia, an unexpected finding given the prevailing view of TAO as a systemic inflammatory condition. Chronic mechanical stimulation, such as that resulting from orbital congestion in TAO, may induce habituation of the oculocardiac reflex—a well-recognized fatigable reflex mediated by the trigeminal (afferent) and vagus (efferent) nerves (Arnold, 2021; Kincaid et al., 2025). This fatigue phenomenon may be associated with blunted vagal responsiveness to acute stimuli, including corticosteroid pulse therapy, which could partly explain the observed lower bradycardia risk. Concurrently, systemic inflammation may suppress vagal outflow, while vagal tone exerts anti-inflammatory effects via the cholinergic pathway—suggesting a bidirectional interplay that may modulate bradycardia susceptibility (Williams et al., 2019; Hilderman and Bruchfeld, 2020). These findings suggest a potential association between ocular symptoms and bradycardia risk, which may inform individualized consideration in TAO management. However, given the exploratory nature of our findings, this hypothesis requires prospective validation. Nevertheless, our data support the need for careful HR monitoring, particularly during IVMP therapy in patients without periorbital edema.

In this study, concomitant cardiac disease—including atrioventricular block, premature ventricular contraction, coronary heart disease, and rheumatic heart disease—was independently associated with a substantially higher likelihood of sinus bradycardia after IVMP therapy (HR 2.454, 95% CI 1.105–5.450, *p* < 0.05). Consistent with this, the 2018 ACC/AHA/HRS Guideline and the literature review also recognize these as related factors for bradycardia (Kusumoto et al., 2019; Sidhu and Marine, 2020). Although the precise mechanisms underlying bradycardia during high-dose corticosteroid therapy in patients with pre-existing cardiac disease remain incompletely defined, several potential mechanisms involving cardiac electrophysiology and autonomic regulation have been proposed (as discussed in Section 4.4), many of which may be amplified in the presence of underlying heart disease. Therefore, for patients with heart diseases, a thorough assessment should be conducted before treatment, and HR should be closely monitored afterward, with treatment measures implemented as needed.

Although baseline median TSH, FT3, and FT4 levels were within the normal reference ranges in both groups, higher TSH within the euthyroid range was significantly associated with an increased likelihood of sinus bradycardia. This finding suggests that even subtle variations in thyroid function may exert measurable effects on cardiac autonomic regulation (Casu et al., 2005; Aktar et al., 2024), particularly under acute stressors such as high-dose corticosteroid therapy. Given that FT3 and FT4 levels did not differ between groups, the observed association may reflect inter-individual differences in hypothalamic-pituitary-thyroid axis set point or tissue-level thyroid hormone sensitivity, rather than overt differences in circulating hormone levels (Astapova et al., 2011; Leow and Goede, 2014). Thus, TSH variability may serve as a sensitive integrative marker of cardiac autonomic reserve, with clinical relevance emerging only under conditions of physiological challenge.

β-Blockers are commonly used to relieve systemic sympathetic symptoms in patients with hyperthyroidism, such as tachycardia and hand tremors. These agents competitively antagonize cardiac β1-receptors, thereby inhibiting the sympathetic nervous system-mediated increase in HR and myocardial contractility (Schultz et al., 2025). As a result, concurrent β-blocker use may be associated with a higher rate of bradycardia in patients receiving IVMP. Supporting this, Yong et al. found that among patients with thyroid eye disease receiving IVMP therapy, those also on β-blockers exhibited a higher propensity for bradycardia (Yong et al., 2015). However, the present study did not identify β-blocker use as an independently associated factor, possibly owing to the limited sample size.

### 4.4 Potential mechanisms underlying IVMP-induced bradycardia

Although the exact biological mechanisms underlying bradycardia induced by IVMP therapy remain incompletely understood, several hypotheses have been proposed based on current evidence, and the condition is generally considered multifactorial in nature. The most well-established mechanism is central baroreflex resetting. Bechtold and Scheuer demonstrated in conscious rats that corticosterone acts directly in the dorsal hindbrain, which encompasses the nucleus tractus solitarius (NTS), to increase the arterial pressure midpoint of the HR baroreflex from 108 to 123 mmHg and to reduce the maximum gain from 3.51 to 2.72 beats/min/mmHg^-1^ (Bechtold and Scheuer, 2006; Scheuer, 2009). This resetting is pressure-independent and active, rather than passively following changes in baseline arterial pressure, suggesting that glucocorticoids modify the central set point for HR regulation at the level of the NTS.

Another proposed mechanism involves volume expansion and baroreceptor activation. Glucocorticoids may promote sodium and water retention, increasing blood volume and arterial pressure, thereby triggering baroreceptor-mediated reflex bradycardia. However, direct supporting evidence for this hypothesis remains limited. In fact, Scheuer demonstrated that localized dorsal hindbrain corticosterone administration can modulate baroreflex function without altering plasma corticosterone levels (Scheuer, 2009). In our study, BP remained normal or even decreased at the time of bradycardia, challenging the clinical relevance of this proposed mechanism. Additionally, animal studies have suggested that corticosteroids may suppress cardiovascular β1-receptor sensitivity (Zhang et al., 2022).

Alterations in autonomic tone have also been implicated. Glucocorticoids may suppress sympathetic efferent outflow or enhance vagal tone, shifting cardiac autonomic balance toward parasympathetic predominance. Scheuer and Mifflin showed that systemic corticosterone treatment modulates baroreflex control of renal sympathetic nerve activity (Scheuer and Mifflin, 2001), and Scheuer further suggested that NTS catecholaminergic neurons may mediate these central autonomic effects (Scheuer, 2009).

Finally, electrolyte transmembrane shifts represent another theoretical mechanism. High-dose glucocorticoids may exert mineralocorticoid-like effects, influencing myocardial membrane permeability to potassium and sodium ions, thereby disturbing cardiac electrophysiological activity (Kumar et al., 2024). Clinical and epidemiological evidence suggests that magnesium supplementation can often reverse this condition (Chrysant and Chrysant, 2019). Notably, both the previous studies (Zkib et al., 2024; Das et al., 2024) and our own observations have documented normal serum electrolyte levels at the time of bradycardia, casting doubt on the clinical relevance of this pathway.

In summary, while central baroreflex resetting in the NTS appears to be the primary upstream mechanism, the roles of volume expansion, autonomic imbalance, and electrolyte disturbances need further study. The multifactorial nature of IVMP-induced bradycardia underscores the need to identify individual risk factors—cardiac disease, baseline HR, primary disease severity, autonomic dysfunction, and BMI—to guide prevention and management.

## 5 Limitations

Several limitations of this study should be acknowledged. First, the retrospective single-center design and the relatively small number of bradycardia events (n = 48) limited our statistical power and constrained the number of covariates that could be reliably entered into the multivariable model, raising the risk of overfitting. To mitigate this, we restricted adjustment to the most clinically relevant covariates and conducted sensitivity analyses using nested Cox regression models, which yielded consistent results. Nonetheless, the modest discriminative performance (C-index 0.605) suggests limited utility for individual risk stratification and warrants cautious interpretation.

Second, the observational nature of the study precludes any definitive causal inferences. Accordingly, our findings should be regarded as exploratory and hypothesis-generating, requiring external validation in independent cohorts before clinical application can be considered.

Third, despite adjustment for multiple clinically relevant confounders, we cannot entirely rule out residual or unmeasured confounding—including concomitant use of cardioactive medications (e.g., calcium channel blockers or other negative chronotropic agents), coexisting conditions (e.g., hypertension, diabetes, or dyslipidemia), and individual susceptibility—which may have influenced the observed associations.

Finally, the generalizability of our findings may be limited by the single-center setting and the relatively homogeneous study population. Thus, large-scale, prospective, multicenter studies are warranted to confirm our observations and further elucidate the underlying mechanisms.

## 6 Conclusion

This study demonstrates that IVMP therapy is associated with bradycardia in approximately 30% of TAO patients. The mean lowest HR during the first bradycardic episode was 46.9 beats/min, representing a 42.1% reduction from baseline. Nearly 80% of affected patients experienced their first episode within 3 days of treatment initiation, with onset ranging from 1.3 h to 12 days. Most cases of sinus bradycardia resolved spontaneously without intervention. Cox regression analysis revealed that cardiac disease and TSH were independently positively associated with bradycardia, whereas HR, BMI, and periorbital edema were independently negatively associated with it. These findings suggest that enhanced surveillance and appropriate management of bradycardia may be warranted in high-risk patients.

## Data Availability

The original contributions presented in the study are included in the article, further inquiries can be directed to the corresponding author.
